# Mobile Crisis Outreach and Emergency Department Utilization: A Propensity Score-matched Analysis

**DOI:** 10.5811/westjem.2021.6.52276

**Published:** 2021-09-02

**Authors:** J. Priyanka Vakkalanka, Ryan A. Neuhaus, Karisa K. Harland, Lance Clemsen, Elaine Himadi, Sangil Lee

**Affiliations:** * University of Iowa Carver College of Medicine, Department of Emergency Medicine, Iowa City, Iowa; † University of Iowa College of Public Health, Department of Epidemiology, Iowa City, Iowa; ‡ University of Iowa Carver College of Medicine, Department of Psychiatry, Iowa City, Iowa

## Abstract

**Introduction:**

Mental health and substance use disorder (MHSUD) patients in the emergency department (ED) have been facing increasing lengths of stay due to a shortage of inpatient beds. Previous research indicates mobile crisis outreach (MCO) reduces long ED stays for MHSUD patients. Our objective was to assess the impact of MCO contact on future ED utilization.

**Methods:**

We conducted a retrospective chart review of patients presenting to a large Midwest university ED with an MHSUD chief complaint from 2015–2018. We defined the exposure as those who had MCO contact and any MHSUD-related ED visit within 30 days of MCO contact. The MCO patients were 2:1 propensity score–matched by demographic data and comorbidities matched to patients with no MCO contact. Outcomes were all-cause and psychiatric-specific reasons for return to the ED within one year of the index ED visit. We report descriptive statistics and odds ratios (OR) to describe the difference between the two groups, and hazard ratios (HR) to estimate the risk of return ED visit.

**Results:**

The final sample included 106 MCO and 196 non-MCO patients. The MCO patients were more likely to be homeless (OR 14.8; 95% confidence interval [CI],1.87, 117), less likely to have adequate family or social support (OR 0.51; 95% CI, 0.31, 0.84), and less likely to have a hospital bed requested for them in the index visit by ED providers (OR 0.50; 95% CI, 0.29, 0.88). For those who returned to the ED, the median time for all-cause return to the ED was 28 days (interquartile range [IQR]: 6–93 days) for the MCO patients and 88 days (IQR: 20–164 days) for non-MCO patients. The risk of all-cause return to the ED was greater among MCO patients (67%) compared to non-MCO patients (49%) (adjusted HR: 1.66; 95% CI, 1.22, 2.27).

**Conclusion:**

The MCO patients had less family and social support; however, they were less likely to require hospitalization for each visit, likely due to MCO involvement. Patients with MCO contact presented to the ED more frequently than non-MCO patients, which implies a strong linkage between the ED and MCO in our community. An effective referral to community service from the ED and MCO and collaboration could be the next step to improve healthcare utilization.

## INTRODUCTION

It is estimated that one in every eight emergency department (ED) visits in the United States is related to mental health and/or substance use disorders (MHSUD).^1^,^2^ Limited numbers of inpatient psychiatric beds force many patients with MHSUDs to stay in the ED for an extended time for placement and reassessment before release.^3^–^5^ This practice has the potential to affect the quality of care provided to patients with MHSUDs, increases ED crowding, places greater demands on emergency care providers, and leads to longer wait times for other ED patients. This demonstrates the need for greater community services to support this population.^6^

Mobile crisis outreach (MCO) is a community outreach program that provides de-escalation, support, assessment, and future safety planning for those with mental health concerns.^7^ The MCO providers can engage patients in the community to help ensure the patient’s safety and the safety of other community members, while working to reduce the number of MHSUD patients inappropriately presenting to the ED.^8^ Research has shown that MCO programs can be cost-effective, reduce costs associated with hospitalization and readmissions, and reduce expenses in the criminal justice system.^7^ Additionally, MCO services are sometimes used to connect MHSUD patients to stabilization services following discharge from the ED.^9^ Although several impacts have been reported, the effectiveness of the MCO and its effect on healthcare utilization in rural America has not been well reported. The role of the MCO could be very different for those who reside in rural area, as resources can be limited.

Intervention by a MCO in a crisis may reduce the number of patients with an MHSUD presenting to the ED, because MCO providers may be able to de-escalate the current crisis and connect the patient to community mental health services.^8^ The objective of this study was to evaluate the effectiveness of an MCO program in a rural Midwestern county. We hypothesized that access to MCO services is associated with decreased ED utilization, psychiatric hospitalization, and suicide-related death.

## METHODS

### Study Design

This study was a retrospective, propensity-score matched cohort study of patients presenting to a Midwestern ED between January 1, 2015–December 31, 2018. The study was approved by the local institutional review board under waiver of informed consent, and this article is in accordance with the STrengthening the Reporting of OBservational studies in Epidemiology (STROBE) statement.^10^

### Data Sources

Data sources included the health system’s electronic health records (EHR) and the call records from the local MCO. The study took place at an academic institution that provides tertiary care and has access to psychiatry service covering the majority of Johnson County, Iowa, and has an annual ED census of 60,000. Data from the hospital system’s EHR included demographic and clinical information regarding the patients.

The MCO program of Johnson County provides a coverage of 600 square miles to a population of about 150,000, by a 24/7 telephone hotline and dispatch service by two trained mental health professionals in a vehicle to a public place or private residence to aid those in need. A request for MCO may be initiated by the patient, family or caregiver, law enforcement, and healthcare provider. The goals of MCO are to stabilize the crisis, assess the need for referrals to other community services, reduce unnecessary hospitalizations and arrests, and admit the client to a crisis stabilization bed when clinically indicated. The referral to MCO occurred during or after ED visits, or the MCO program referred its clients to any EDs in the region (a community ED also covers Johnson County); however, from reviewing the MCO call log for ED referrals, we found that 80% of MCO cases presented to our ED during the study period from directly linking the two data sources. Information from the MCO call log indicated patient identification information, such as name, date of birth, and address, which we used to link to the hospital EHR.

**Population Health Research Capsule**

What do we already know about this issue?

*Mobile crisis outreach (MCO has been used for crisis stabilization when patients with mental health conditions and substance use disorder (MHSUD) seek care in the ED*.

What was the research question?

*Is the MCO program associated with a change in all-cause and psychiatric-related ED visits?*

What was the major finding of the study?

*The MCO users had decreased hospitalizations but increased ED visits for all reasons and MHSUD reasons compared to non-MCO users*.

How does this improve population health?

*Access to a MCO program could be key to stabilizing a crisis situation in the community. The linkage between such an outreach program and the ED needs to be optimized*.

We accessed state death registry data to identify any fatality within one year after the index ED visit for both the MCO and non-MCO groups.

### Study Population

We defined exposure group as patients who received MCO services, as identified from the MCO call log, and who presented to the ED ±30 days of the MCO contact (39% ED first, 35% MCO first, and 26% same day) with an MHSUD diagnosis. We chose 30 days to consider two possible scenarios in the sequence of events. First, an MCO exposure could have occurred first, which subsequently led to a referral to be seen in the ED for additional care. Second, an ED visit could have occurred first, and the MCO was identified for follow-up upon discharge from the ED. Then, we conducted manual record review to identify a matching patient in our EHR who was listed in the MCO call log.

We included those who were classified as having an MHSUD diagnosis if any diagnosis code matched to the Clinical Classification Software (CCS) Level 1 codes indicating “Mental Health” (CCS Level 1 code of “5”) using the International Classification of Diseases, 9^th^ and 10^th^ editions (ICD-9 and ICD-10).^11^ We additionally used the CCS Level 2 codes to classify other select psychiatric comorbidities that most frequently occurred within this sample including anxiety (5.2), mood disorders (5.8), suicide (5.13), and schizophrenia (5.10) using the CCS v2019.1 (beta version) files available from the Healthcare Cost and Utilization Project tools. These frequently occurring diagnostic codes are presented in Appendix A. The MCO-exposed individuals were then matched to unexposed patients (ie, non-MCO) in up to a 2:1 propensity-score match on demographics and the psychiatric diagnoses diagnosis from the EHR.

### Measurement of Primary Exposure

The primary exposure of interest was any MCO contact. Patients who were seen in the ED but did not have any MCO contact were considered unexposed (non-MCO). A patient’s MCO status was identified deterministically by verifying the patient’s unique name, date of birth, and address from the MCO call list and EHR.

### Measurements of Covariates and Potential Confounders

We obtained covariate data from administrative data files within the health system and manual data extraction from the ED chart at the time of the visit. Specifically, administrative data included demographic variables such as gender, age (<18, 18–34, 35–49, and >50 years), insurance (commercial, Medicaid, Medicare, and other such as military), and other co-morbidities documented as diagnostic codes from the EHR. Manually extracted covariate data from the ED visit included documentation of situational characteristics such as access to firearms, whether the patient was homeless or from a residential facility, had adequate family or social support based on the documentation of such support from providers and social workers, and was accompanied to the ED by another individual (family/friend, law enforcement, MCO, or other third party such as a social worker or teacher). We also determined healthcare access and utilization from chart review and included contact with the ED, a primary care provider, and psychiatry provider within the 12 months prior to the ED visit. Disposition from the ED was characterized by whether or not a bed request for any inpatient admission had been placed in the ED. We also included chief complaints and current medication list at the time of the ED visit.

### Key Outcome Measures

The primary outcome in this study was time to all-cause return to the ED within one year (365 days) of the ED visit. We manually coded the return ED visit as psychiatric, suicide, medical, overdose (intoxication), or surgical (non-injury), and allowed for multiple choices. One year was chosen as the outcome to capture a rare outcome such as completed suicide. If the patient had multiple visits within 365 days of the index visit, time in days to the first ED visit was used. As a secondary outcome, we assessed time to return to the ED for an MHSUD-related visit if it included psychiatric, suicide, or overdose. We also accessed the State of Iowa death registry to identify any death and cause of death including suicide within one year of the ED visit.

### Statistical Analysis

This analysis was conducted using time-to-event analyses of propensity-matched pairs. We analyzed covariates (clinical histories, presentation, and medications) obtained from medical chart review on the association with MCO and return to the ED for any reason.

### Sample Size Calculation

To determine the appropriate sample size given a set number of patients who received MCO in the study period (N = 170), we used the return-to-ED proportion found in our previous work of 41.7% for MCO-positive (MCO+) patients and determined a priori that a 15% difference in the non-MCO group (56.7%) was clinically important. With 82.5% power and an alpha of 0.05 this resulted in a two non-MCO to one MCO match.^4^

### Propensity Score Matching

The MCO patients were matched to up to two non-MCO patients through optimal matching propensity score (PS) methodology (Table 1).^12^,^13^ All MCO patients had at least one control, although two controls were not identified for every MCO patient due to a limited pool of non-MCO patients. We determined a priori that all available variables in the administrative dataset would be used in the PS model. As a result, we calculated the PS for MCO utilization as an outcome based on three demographic variables (age, gender, and insurance) and several MHSUD-related conditions from diagnostic codes such as anxiety disorders, mood disorders, suicide, and substance dependence. We evaluated whether the confounders were balanced across PS-matched pairs using standardized differences and determined a priori that unbalanced covariates (with a standardized difference greater than 0.1) would be assessed for inclusion in final regression models.

### Evaluation of Covariates

Within the matched cohort, we measured differences in the clinical histories, comorbidities, and presenting characteristics (obtained from chart extraction) between the MCO and non-MCO cohorts. Bivariate analyses for the association between MCO and each covariate were conducted using conditional logistic regression to determine unadjusted odds ratios (uOR) for each matched pair for binary outcomes, clustered on match ID. Those variables associated with MCO were later considered in developing final multivariable models for the time-to-event analyses of return to the ED.

### Evaluation of Outcomes

We assessed Kaplan-Meier survival curves to estimate the time-to-event for both all-cause and psychiatric-specific return to the ED by MCO status. Log-rank tests were used to compare probabilities of survival by MCO status. The proportional hazards assumptions were assessed by evaluation of the negative log and log of the negative log survival plots. We evaluated the adjusted hazard ratios (aHR) of return to the ED through the Cox proportional hazards frailty model, clustering on the matched pair identified from the propensity score.

### Final Multivariable Models

The final multivariable models for the association between MCO status and all-cause and psychiatric-specific return to the ED were evaluated for potential confounding by purposeful selection. We included all covariates that were associated with the exposure status and the outcome from bivariate analyses, and those that were not balanced after PS matching. Variables were removed if they were not independently associated with the outcome in the adjusted model or did not significantly affect the MCO exposure measure. We additionally calculated E-values in sensitivity analyses of both outcomes to assess the potential effect of unmeasured confounders.^13^ All tests were considered significant at alpha <0.05 using two-tailed tests. We completed analyses using SAS version 9.4 (SAS Institute Inc, Cary, NC).

### Demographics and Characteristics of Population

**Figure 1:**
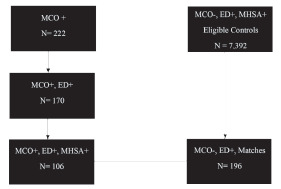
Flowchart of sample selection.

Of the 222 patients who were identified from the MCO call log, 106 were seen in the ED for a MHSUD complaint (Figure 1). The final study sample included 302 patients (n = 106 MCO exposure patients, and n = 196 non-MCO exposure patients). The two cohorts were balanced by gender and mental health comorbidities, which included any diagnosis from the index ED visit after PS matching (Table 1).

### Situational Factors and Clinical Presentation

At the index ED visit, the proportion of homelessness was greater in MCO+ patients (uOR 14.8; 95% CI, 1.87, 117) (Table 2). The odds of reporting adequate family or social support and being accompanied to the ED by a family member or friend were lower in MCO patients compared to non-MCO patients (uOR 0.51; 95% CI, 0.31, 0.84 and uOR 0.32; 95% CI, 0.16, 0.64, respectively). At the ED index visit, MCO patients presented less frequently with overdose (uOR 0.33; 95% CI, 0.11, 0.97) but presented more frequently for suicidal ideation/attempt (uOR 3.09; 95% CI, 1.47, 6.51). Analysis showed that suicide attempts involved nine (7.2%) cases of overdose.

There was no difference in seeing a primary care provider between MCO and non-MCO patients, but MCO patients more often had contact with a psychiatry provider within the 12 months preceding the ED visit (OR 2.09; 95% CI, 1.22, 3.57). The MCO patients were less likely to have a hospital bed requested for them in the index visit by emergency care providers (OR 0.50; 95% CI, 0.29, 0.88).

### Primary Outcome: All-cause Return to the ED

**Figure 2:**
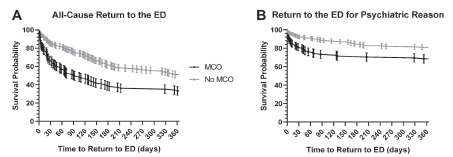
Survival curves of return to the emergency department by mobile crisis outreach exposure status. *ED*, emergency department; *MCO*, mobile crisis outreach.

Among patients returning to the ED, the median time for all-cause return was 28 days (IQR range: 6–93 days) for the MCO patients and 88 days (IQR: 20–164) for non-MCO patients. In the final multivariable model adjusting for the presence of family support, the risk of all-cause return to the ED was greater among MCO patients (67%) compared to non-MCO patients (49%) (aHR: 1.66; 95% CI, 1.22, 2.27) (Table 3, Figure 2). In the sensitivity analysis of interpreting the E-value, the observed HR of 1.66 could be explained away by an unmeasured confounder that was associated with both the treatment and the outcome by a HR of 2.71-fold each, above and beyond the measured confounders, but weaker confounding could not do so.

### Secondary Outcome: Psychiatric Reason for Return to the Emergency Department

Among patients returning to the ED for psychiatric reasons, the median time for return to the ED was 17 days (IQR: 4–54 days) for the MCO patients and 64 days (IQR: 13–164) for non-MCO patients. The MCO exposure was associated with return to the ED (*P* <0.001). In the final multivariable model adjusting for the presence of previous visit for suicidal ideation/attempt, bed request at index visit, and schizophrenia, the risk of return to the ED for psychiatric reasons was greater among MCO patients compared to non-MCO patients (aHR: 1.70; 95% CI, 1.06, 2.74) (Table 3, Figure 2). In the sensitivity analysis of interpreting the E-value, the observed HR of 1.70 could be explained away by an unmeasured confounder that was associated with both the treatment and the outcome by a HR of 2.79-fold each, above and beyond the measured confounders, but weaker confounding could not do so.

### Secondary Outcome: Mortality

There were four deaths due to natural causes and one death due to suicide in this cohort within one year of the index visit, all of which occurred in the non-MCO group; however, there was no statistically significant difference in the rate of death and suicide-related death between the two groups (*P* = 0.166).

## DISCUSSION

Our study demonstrated the unique characteristics of patients who used the MCO program during the study period, such as higher rates of homelessness and limited family support. The use of MCO was associated with a decreased risk of hospitalization during the index ED visit. It also demonstrated increased ED utilization for any and psychiatric-specific reasons compared to those who did not use the MCO service. The proportion of deaths was not statistically significantly different between the two groups, although the sample size was likely too small to detect potential differences. Patients who had received MCO services were more likely to be homeless and less likely to have adequate social support than the control cohort. The proportion of homelessness in the MCO group was similar to that reported previously by Scott et al.^7^ Another study showed the effectiveness of MCO for the homeless population.^15^ Perhaps patients in our study who had MCO contact may have also been using MCO and ED resources to fulfill their social support needs, such as housing, day care, and shelter.

Use of MCO services was associated with fewer bed requests made by ED providers. Our rates of MCO patient hospitalization were similar to those reported by Guo et al.^16^ Reductions in hospitalization with the use of MCO were found by Guo et al and Hugo et al.^16^, ^17^ Fisher et al found no difference in psychiatric admission rates between communities that provided mobile crisis services and those that did not.^18^ The disparity in results is likely due to differences in study populations. The methodology used by Guo et al and Hugo et al was similar to what we used in our study in that they compared hospitalization rates between patients who used MCO services with those who did not, while Fisher et al compared patients who had MCO services in their communities to those who did not have community-based MCO services.^16^–^18^ We speculate that the ED used the MCO when a patient needed an alternative disposition other than hospitalization.

Patients who received MCO services were more likely to return to the ED for all causes and for psychiatric causes. Currier et al reported that the MCO group continued to experience persistent symptoms and risk for return visits.^9^ Fendrich et al found that youths who received MCO services had decreased odds of having a behavioral health ED visit.^19^ How MCO services may differentially affect adults vs youths is unknown. Our MCO program was focused on adult patients who had more limited family support, and that focus may have led to the increase in reported return visits. Our study finding demonstrated the MCO led to a referral to a higher level of care, in this case, to the ED. It also elucidated that while the crisis at the index ED visit was mitigated, post-MCO usage and post-index ED visits care need further improvement for this vulnerable population. Continued contact or follow-up with these individuals may perhaps be necessary to ensure they are appropriately and adequately navigating the healthcare system to meet their healthcare needs.

Our study used a PS score to balance the prognosis of ED return visits between MCO patients and non-MCO patients. Because the PS matching procedure cannot account for unmeasured confounders, we used E-value to estimate a confounder’s role that could have led to our study conclusion.^20^ We evaluated many risk factors associated with ED return visits, as reported in the previous study.^21^ Most of the predictors were accounted for in this study, including frequent ED utilization status, which reported an OR of 5.6.^21^ Thus, concerns about validity or the extent of unmeasured confounding were mitigated in our assessment of the impact of MCO exposure on future ED utilization.

The rate of mortality remained small in both MCO and non-MCO groups in our study. This is also similar to the finding in our previous study, where patients were reassessed and released after an ED provider or psychiatrist recommended hospitalization at the initial evaluation.^4^ The study was likely underpowered to detect any significant difference, but this is still vital knowledge to share, as a completed suicide is a devastating outcome for those discharged from the ED. The one case of suicide death in our sample was a middle-aged male brought to the ED for a suicide attempt by running a car in a closed garage. Approximately three weeks after the index ED visit, he died by suicide with a discharge of a homemade, low-explosive device in or around the oral cavity. Effective prevention of suicide remains a key challenge in ED psychiatric research.

## LIMITATIONS

Our study has several limitations. First, it is a retrospective review of MCO contact and ED visits at a single hospital. Both MCO and non-MCO patients may have had ED visits at other hospitals within the year time frame that were not captured. The previous data showed that about 80% of MCO patients were referred to our institution when the MCO determined that a referral to the ED was needed. Second, given the observational study design, patients were not randomly assigned to the MCO and, therefore, there may be unmeasured confounding. To overcome this limitation we used propensity score methodology to create cohorts balanced on administrative characteristics; the cohorts were balanced following the PS match, reducing the likelihood of unmeasured confounding, and we added the component of the sensitivity analysis by introducing E-value. Third, the linkage between the ED utilization and the MCO exposure and matching procedure led to the loss of significant samples. Fourth, we used subjective rating of family and social support, so the rating could be prone to bias.

## CONCLUSION

The mobile crisis outreach program has served as an alternative resource in the community for those with mental health/substance use disorders, and it shows a reduction of hospitalization but an increase in subsequent ED utilization. In the setting of constrained inpatient resources, the use of the MCO may be a reasonable alternative for those who present to the ED or those who have a crisis situation to benefit from assessment before ED referral. A strong linkage between the MCO program, ED, and outpatient resources is necessary to sustain high-quality mental healthcare, particularly after the MCO access and index ED visits.

## Supplementary Information



## Figures and Tables

**Table 1:** Comparison of demographics between cohorts receiving and not receiving mobile crisis outreach consultation.

Charactistics	Total N=302	MCO N=106		No MCO N=196		Standardized Difference

	N	N	%	N	%	
Demographics						
Female	137	50	(47.2)	87	(44.4)	0.06
Age						
< 18	19	7	(6.6)	12	(6.1)	0.02
18–34	131	42	(39.6)	89	(45.4)	−0.12
35–49	73	30	(28.3)	43	(21.9)	0.15
>50	79	27	(25.5)	52	(26.5)	−0.02
Insurance						
Commercial	134	20	(18.9)	114	(58.2)	−0.88
Medicaid	44	44	(41.5)	0	(0.0)	N/A
Medicare	86	23	(21.7)	63	(32.1)	−0.24
Other	38	19	(17.9)	19	(9.7)	0.24
Comorbidities						
Anxiety disorders	58	20	(18.9)	38	(19.4)	−0.01
Mood disorders	28	10	(9.4)	18	(9.2)	0.01
Substance dependence	32	18	(17.0)	14	(7.1)	0.31
Suicidal ideation	125	47	(44.3)	78	(39.8)	0.09
Schizophrenia	41	13	(12.3)	28	(14.3)	−0.06

*MCO*, mobile crisis outreach; *MHSUD*, mental health and substance use disorder.

**Table 2:** Comparison of situational, clinical characteristics, and healthcare access and follow-up between cohorts with and without exposure to mobile crisis outreach.

Characteristics		MCO Exposure (N=106)		Non-MCO Exposure (N=196)		uOR	95% CI

	N	n (%)		n (%)			
Situational Characteristics							

Access to firearms	18	7	(39)	11	(61)	1.23	0.42–3.66
Homeless	12	10	(83)	2	(17)	14.8	1.87–117.12
From residential facility	40	18	(45)	22	(55)	1.58	0.81–3.08
Adequate family or social support*	155	43	(28)	112	(72)	0.51	0.31–0.84
Accompanied to ED by:							
Family/Friend*	94	21	(22)	73	(78)	0.32	0.16–0.64
Law enforcement	40	14	(35)	26	(65)	0.98	0.46–2.07
MCO	45	45	(100)	0	(0)	--	--
Other third party (eg, social worker, teacher)	16	7	(44)	9	(56)	1.56	0.58–4.18
None	122	38	(31)	84	(69)	0.7	0.41–1.19
Unknown	12	2	(17)	10	(83)	0.23	0.03–1.91

Healthcare Access and Follow-up							

Bed requested by ED (vs discharged)*	116	31	(27)	85	(73)	0.5	0.29–0.88
≥1 ED visit in past 12 months*	161	72	(45)	89	(55)	2.48	1.50–4.09
Regular follow-up by primary care provider	165	60	(36)	105	(64)	1.14	0.69–1.87
Regular follow-up by primary psychiatry provider*	98	44	(45)	54	(55)	2.09	1.22–3.57
Chief Complaints #							
Agitation/Altered mental status	12	2	(17)	10	(83)	0.35	0.07–1.73
Bipolar disorder	8	4	(50)	4	(50)	2.26	0.49–10.42
Depression	131	47	(36)	84	(64)	1.15	0.65–2.02
Hallucinations/Delusions	45	14	(31)	31	(69)	0.78	0.35–1.75
Injury	8	4	(50)	4	(50)	2	0.50–8.00
Overdose* (intentional and non-intentional)	32	7	(22)	25	(78)	0.33	0.11–0.97
Suicide* (suicidal ideation and attempt)	147	60	(41)	87	(59)	3.09	1.47–6.51

Current Medications							

Antidepressants	127	50	(39)	77	(61)	1.4	0.86–2.27
Antipsychotics	79	27	(34)	52	(66)	0.96	0.53–1.73
Anxiolytics (benzodiazepines, non-benzodiazepines)	85	35	(41)	50	(59)	1.44	0.85–2.45
Drugs for substance use disorder	14	4	(29)	10	(71)	0.66	0.20–2.21
Hypnotics*	64	31	(48)	33	(52)	2.09	1.18–3.71

* indicates significant result; # multiple entries allowed.

*MCO*, mobile crisis outreach; *uOR*, unadjusted odds ratio; *CI*, confidence interval; *ED*, emergency department.

**Table 3:** Association between exposure to mobile crisis outreach and return to the emergency department.

Outcome	MCO N (%)	Non-MCO N (%)	uHR	95%CI	aHR	95% CI
Any Return to ED^1^ (N=167)	71 (67)	96 (49)	1.75	1.29, 2.38	1.66	1.22, 2.27
Any Psych-Related Return to ED^2^ (N=72)	34 (32)	38 (19)	1.87	1.18, 2.97	1.70	1.06, 2.74

1 Adjusted for indicator of family support.

2 Adjusted for schizophrenia, bed request at index visit, and previous visit for suicidal ideation.

*MCO*, mobile crisis outreach; *uHR*: unadjusted odds ratio; *aHR*, adjusted hazard ratio; *N*, number of patients; *CI*, confidence interval; *ED*, emergency department.

## References

[1] Rui P, Kang K, Ashman JJ (2016). National Hospital Ambulatory Medical Care Survey.

[2] Owens PL, Mutter R, Stocks C (2007). Mental health and substance abuse-related emergency department visits among adults, 2007.

[3] Wharff EA, Ginnis KB, Ross AM (2011). Predictors of psychiatric boarding in the pediatric emergency department: implications for emergency care. Pediatr Emerg Care.

[4] Lee S, Harland KK, Swanson MB (2018). Safety of reassessment-and-release practice for mental health patients boarded in the emergency department. Am J Emerg Med.

[5] Nesper AC, Morris BA, Scher LM (2016). Effect of decreasing country mental health services on the emergency department. Ann Emerg Med.

[6] Derlet RW, Richards JR (2000). Overcrowding in the nation’s emergency departments: complex causes and disturbing effects. Ann Emerg Med.

[7] Scott RL (2000). Evaluation of a mobile crisis program: effectiveness, efficiency, and consumer satisfaction. Psychiatr Serv.

[8] Simakhodskaya Z, Haddad F, Quintero M (2009). Innovative use of crisis intervention services with psychiatric emergency room patients. Prim Psychiatry.

[9] Currier GW, Fisher SG, Caine ED (2010). Mobile crisis team intervention to enhance linkage of discharged suicidal emergency department patients to outpatient psychiatric services: a randomized controlled trial. Acad Emerg Med.

[10] von Elm E, Altman DG, Egger M (2007). The Strengthening the Reporting of Observational Studies in Epidemiology (STROBE) statement: guidelines for reporting observational studies. BMJ.

[11] Agency for Healthcare research and Quality (2015). Healthcare Cost and Utilization Project Clinical Classification Software.

[12] SAS Institute Inc (2016). SAS/STAT 14.2 User’s Guide - The PSMATCH Procedure.

[13] Austin PC (2011). An introduction to propensity score methods for reducing the effects of confounding in observational studies. Multivariate Behav Res.

[14] WanderWeele TJ, Ding P (2017). Sensitivity analysis in observational research: introducing the E-value. Ann Intern Med.

[15] Morris DW, Warnock JK (2000). Effectiveness of a mobile outreach and crisis services unit in reducing psychiatric symptoms in a population of homeless persons with severe mental illness. J Okla State Med Assoc.

[16] Guo S, Biegel DE, Johnsen JA (2001). Assessing the impact of community-based mobile crisis services on preventing hospitalization. Psychiatr Serv.

[17] Hugo M, Smout M, Bannister J (2002). A comparison in hospitalization rates between a community-based mobile emergency service and a hospital-based emergency service. Aust N Z J Psychiatry.

[18] Fisher WH, Geller JF, Wirth-Cauchon J (1990). Empirically assessing the impact of mobile crisis capacity on state hospital admissions. Community Ment Health J.

[19] Fendrich M, Ives M, Kurz J (2019). Impact of mobile crisis services on emergency department use among youths with behavioral health service needs. Psychiatr Serv.

[20] Lee S, Herrin J, Bobo WV (2017). Predictors of return visits among insured emergency department mental health and substance abuse patients, 2005–2013. West J Emerg Med.

[21] Haneuse S, VanderWeele TJ, Arterburn D (2019). Using the E-value to assess the potential effect of unmeasured confounding in observational studies. JAMA.

