# Supplementary material for: Mobile Crisis Outreach and Emergency Department Utilization: A Propensity Score-matched Analysis
Source: West J Emerg Med. 2021 Sep 2;22(5):1086–94. doi: 10.5811/westjem.2021.6.52276 (PMC8463043; doi:10.5811/westjem.2021.6.52276)
Supplement: Supplementary file 1 [file wjem-22-1086-s001.docx]

**Appendix. Most Frequently Identified Psychiatric-Related Diagnostic Codes among MHSUD Patients Presenting to the Emergency Department**

- F20.0 (Paranoid schizophrenia)
- F20.9 (Schizophrenia, unspecified)
- F23 (Brief psychotic disorder)
- F25.9 (Schizoaffective disorder, unspecified)
- F29 (Unspecified psychosis not due to a substance or known physiological condition)
- F31.9 (Bipolar disorder, unspecified)
- F32.9 (Major depressive disorder, single episode, unspecified)
- F33.2 (Major depressive disorder, recurrent severe w/o psych features)
- F41.1 (Generalized anxiety disorder)
- F41.9 (Anxiety disorder, unspecified)
- F43.10 (Post-traumatic stress disorder, unspecified)
- F60.3 (Borderline personality disorder)
- F79 (Unspecified intellectual disabilities)
- F84.0 (Autistic disorder)
- F90.9 (Attention-deficit hyperactivity disorder, unspecified type)
- F91.1 (Conduct disorder, childhood-onset type)
- F91.3 (Oppositional defiant disorder)
- F91.9 (Conduct disorder, unspecified)
- Z91.5 (Personal history of self-harm)
- R45.851 (Suicidal ideations)
- T38, T39, T40, T42, T43, T44, 545, T46, T48, T50, T51, T52 (Poisonings)
- X71, X72, X73, X74, X75, X76, X77, X78, X79, X80, X81, X82, X83 (Suicide and intentional self-inflicted injury)
